# Emerging role of SIRT1 in asthma and COPD from molecular mechanisms to translational therapy

**DOI:** 10.1016/j.isci.2026.115909

**Published:** 2026-04-27

**Authors:** Muhammad Wasim, Rabia Parveen, Riling Chen, Yajun Wang, Guoda Ma

**Affiliations:** 1 Maternal and Children’s Health Research Institute, Shunde Women and Children’s Hospital, Guangdong Medical University, Foshan 528300, China; 2 Department of Pediatrics, Shunde Women and Children Hospital, Guangdong Medical University, Foshan 528300, China; 3 Institute of Pediatrics, Shunde Women and Children’s Hospital, Guangdong Medical University, Foshan 528300, China

**Keywords:** Immune system, Molecular interaction, Non-infectious disease

## Abstract

Asthma and chronic obstructive pulmonary disease (COPD) are chronic respiratory disorders with distinct pathological mechanisms. Even advancements in conventional therapies, the treatment of these diseases remains challenging due to their complex pathophysiology and limited efficacy of current anti-inflammatory treatments. SIRT1, an NAD^+^-dependent deacetylase, exerting protective effects by inhibiting NF-κB and STAT3, activating Nrf2 and FOXO3, and suppressing TGF-β/Smad. In asthma, SIRT1 attenuates Th2 inflammation, mucus hypersecretion, and airway smooth muscle proliferation; in COPD, it reduces neutrophilic inflammation, alveolar senescence, and protease/antiprotease imbalance. Shared benefits include mitigation of oxidative stress, mitochondrial dysfunction, and extracellular matrix remodeling. However, clinical translation faces critical barriers: cell type-specific SIRT1 effects, pharmacokinetic limitations of current activators, and lack of biomarker-guided strategies. This review examines challenges, compares SIRT1’s divergent roles, and evaluates chronotherapy, biomarker-guided selection, and SIRT1 activators. Integration of current evidence and knowledge gaps reveals pathways to personalized SIRT1-targeted therapies for obstructive airway diseases.

## Graphical abstract

**Figure:**
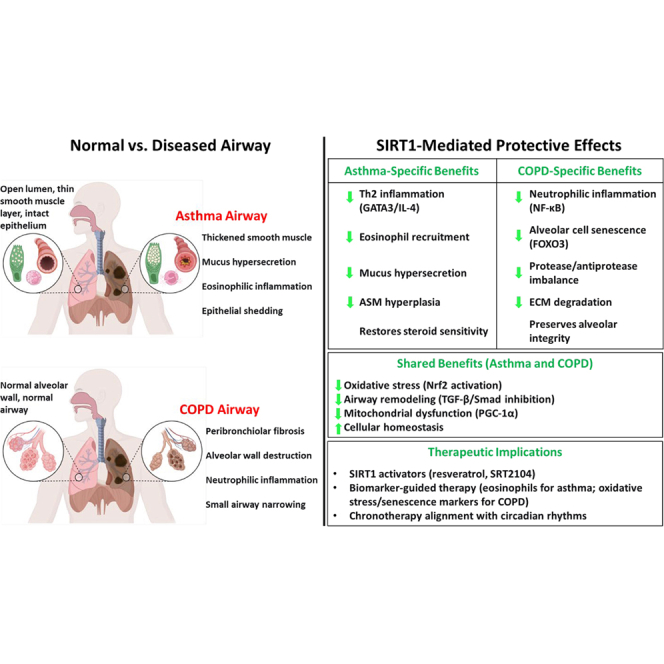

## Introduction

Asthma and chronic obstructive pulmonary disease (COPD) are common chronic respiratory diseases affecting millions of people worldwide, with asthma impacting ∼262 million,^1^ and COPD ∼390 million people.^2^ Prevalence is linked to urbanization, pollution, and lifestyle changes,^3^ both conditions share symptoms like wheezing, coughing, and breathlessness but differ in pathology.^4^ Asthma involves reversible airway inflammation, airway hyperresponsiveness (AHR), and airway remodeling (AR) driven by immune responses and elevated cytokines. COPD, however, features irreversible airflow limitation caused by chronic bronchitis and emphysema, often triggered by smoking and pollutants, leading to inflammation, mucus overproduction, and alveolar damage.^5^^,^^6^ There are several treatment options;^7^^,^^8^ however, early diagnosis and personalized care are vital for better outcomes.^6^^,^^9^^,^^10^

Inflammation and AR are critical pathological features of asthma and COPD, particularly in severe and persistent cases.^11^ AR encompasses structural changes, including smooth muscle hypertrophy, subepithelial fibrosis, and extracellular matrix (ECM) deposition.^12^^,^^13^ These changes are believed to result from repeated cycles of inflammation and repair, driven by the activity of fibroblasts, myofibroblasts, and processes such as epithelial-mesenchymal transition (EMT).^14^^,^^15^^,^^16^ Although AR is an adaptive response to chronic airway injury, it paradoxically induces permanent structural alterations that exacerbate disease severity and reduce responsiveness to conventional anti-inflammatory therapies.^17^ Consequently, targeting the interconnected pathological mechanisms are essential for developing more effective treatments for asthma and COPD,^18^^,^^19^^,^^20^^,^^21^ which required detailed understanding of pathophysiology of both conditions as discussed in the following text.

## Asthma and COPD pathophysiology

Asthma and COPD share features like inflammation and AR but have distinct mechanisms. In asthma, immune cells (eosinophils, Th2/Th17 cells, mast cells, and macrophages) release mediators that perpetuate inflammation.^22^^,^^23^ Critical cytokines, such as IL-4, IL-5, IL-13, TNF-α, and IFN-γ facilitate immune cell recruitment, activation, and IgE production. IgE binds to mast cells, leading to histamine release and triggering bronchoconstriction, mucus secretion, and AHR.^24^^,^^25^ Allergic asthma is driven by Th2-mediated eosinophilic inflammation, causing goblet cell hyperplasia, mucus overproduction, and smooth muscle contraction. In severe steroid-resistant asthma, Th17 cells drive neutrophilic inflammation, worsening AR and reducing treatment response.^26^^,^^27^^,^^28^ Hence, persistent inflammation drives structural changes, including smooth muscle hypertrophy and subepithelial fibrosis, complicating disease management and progression.

Unlike asthma, COPD is characterized by irreversible airflow limitation, which is primarily driven by small AR, mucus hypersecretion, and emphysema. Persistent inflammation and oxidative stress in COPD result in increased production of pro-inflammatory cytokines, such as IL-8, TNF-α, and IFN-γ, which contribute to airway narrowing and lung tissue damage.^29^^,^^30^ Chronic inflammation disrupts protease/antiprotease balance, driving ECM degradation and alveolar destruction in emphysema. Concurrent fibroblast activation and collagen deposition worsen airway fibrosis, causing progressive airflow obstruction.^31^ Various treatment options for managing asthma and COPD are outlined in the following text, but SIRT1 has emerged as a promising therapeutic target due to its regulatory role in mitigating several pathological pathways, offering a potential avenue for improved disease management.

## Treatment options for asthma and COPD

Asthma and COPD have distinct but overlapping treatment approaches.^5^ For asthma, inhaled corticosteroids (ICS) are the primary anti-inflammatory therapy and are often combined with long-acting beta-agonists (LABAs) for moderate to severe cases. ICS reduce airway inflammation, while LABAs improve bronchodilation and symptom relief.^32^^,^^33^ In patients with severe asthma, biologic therapies targeting key inflammatory mediators, such as anti-IgE (omalizumab), anti-IL-5 (mepolizumab and benralizumab), and anti-IL-4/IL-13 (dupilumab), are used to modulate immune responses.^34^^,^^35^^,^^36^ However, short-acting beta-agonists (SABAs) remain first-line agents for acute symptom relief. Non-pharmacological interventions, including patient education, allergen avoidance, and regular monitoring, are crucial components of asthma management.

In COPD management, long-acting muscarinic antagonists (LAMAs) are established as first-line bronchodilators, improving lung function and reducing exacerbations through sustained M3 receptor blockade.^37^ Dual bronchodilation with LAMA/LABA combinations offers synergistic effects via complementary pathways (acetylcholine-mediated bronchoconstriction and β2-adrenergic relaxation) resulting in superior symptom control compared to monotherapy.^38^ In advanced disease, ICS are selectively added to LAMA/LABA regimens, guided by biomarkers such as blood eosinophil counts (≥300 cells/μL) and frequent exacerbation history, to optimize the risk-benefit ratio given ICS-associated pneumonia risk.^39^^,^^40^^,^^41^ Recent evidence confirms that triple therapy (ICS/LAMA/LABA) significantly reduces all-cause mortality and cardiovascular events compared to LAMA/LABA alone,^40^ with the GOLD strategy recommending this personalized approach for patients with eosinophilic phenotypes most likely to benefit.^42^^,^^43^ Systemic corticosteroids and antibiotics may be necessary during exacerbations. Pulmonary rehabilitation, which encompasses exercise training, nutritional support, and breathing techniques, serves as a vital complement to pharmacotherapy, enhancing functional capacity and quality of life in COPD patients.^44^^,^^45^

Emerging therapies, such as phosphodiesterase-4 inhibitors (e.g., roflumilast) for COPD,^46^ and tyrosine kinase inhibitors or SIRT1 activators for both asthma and COPD, show promise in targeting specific pathways involved in AR and inflammation.^47^ Moreover, smoking cessation is a critical intervention in COPD management to slow disease progression, and vaccines against influenza and pneumococcus are recommended to reduce the risk of respiratory infections.^48^ But, advanced cases of COPD may require long-term oxygen therapy or surgical interventions, such as lung volume reduction surgery or lung transplantation. A personalized, stepwise approach to treatment is essential for both conditions, considering disease severity, phenotypes, and patient preferences.^49^^,^^50^

Hence, effective asthma and COPD treatment requires understanding their mechanisms. Our research group has focused on investigating stem cells and the genetic analysis of asthma to elucidate disease mechanisms and enhance treatment efficacy for asthma management.^51^^,^^52^^,^^53^^,^^54^^,^^55^^,^^56^^,^^57^ These studies have revealed that susceptibility genes and stem cell-derived mediators converge on key regulatory pathways including those controlling oxidative stress and inflammation which aligns with the central role of SIRT1 in these processes. Severe asthma needs therapies targeting neutrophilic inflammation, while COPD demands advanced options for irreversible damage. SIRT1 is a promising therapeutic target for both diseases due to its role in regulating vital pathways, offering potential advantages over current treatments. Thus, exploring the specific molecular pathways modulated by SIRT1 is essential to develop personalized treatment strategies for patients.

### Limited clinical trials targeting asthma

Few clinical trials have directly investigated SIRT1 activators (e.g., resveratrol, NAD^+^ boosters) in asthma, with most focusing on COPD or metabolic disorders. For example, a clinical trial of resveratrol in COPD (NCT02245932) showed little anti-inflammatory effects,^58^ but these findings are not directly applicable to asthma due to pathophysiological divergences. SIRT1 activators may differentially modulate Th2 pathways in asthma versus NF-κB-driven inflammation in COPD, necessitating disease-specific trials.^59^ Existing COPD trials also often exclude patients with overlapping asthma, further limiting generalizability.^60^^,^^61^

The role of SIRT1 in Th2 biology is strikingly cell type dependent, acting as either a pro- or anti-inflammatory regulator based on cellular context. In CD4^+^ T cells, SIRT1 functions as a critical suppressor of Th2 responses, as T cell-specific SIRT1 knockout mice exhibit exacerbated allergic inflammation with increased Th2 cell differentiation and cytokine production through the AMPK-mTORC2-SOCS5 axis, which normally restrains IL-4-STAT6-GATA3 signaling.^62^ Mechanistically, SIRT1 directly associates with and destabilizes the Th2 master transcription factor GATA-3, thereby inhibiting GATA-3-induced Th2 cytokine transcription.^63^ Conversely, in dendritic cells (DCs), SIRT1 exerts a pro-Th2 effect by repressing peroxisome proliferator-activated receptor-γ (PPAR-γ) activity, which favors DC maturation toward a pro-Th2 phenotype that promotes allergic airway inflammation; accordingly, pharmacological inhibition or genetic silencing of SIRT1 in DCs derepresses PPAR-γ and dampens Th2 responses.^64^ Adding further complexity, SIRT1 expression in airway epithelial cells suppresses Th2 inflammation during viral-induced asthma exacerbations via the hsa-miR-155-5p-SIRT1 pathway, where targeted inhibition of miR-155-5p blocks exaggerated Th2 inflammation,^65^ while in nasal allergic inflammation models, the TIM4/TIM1 interaction promotes Th2-cell proliferation through enhancing SIRT1 expression via PI3K/Akt phosphorylation.^66^ Collectively, these findings demonstrate that SIRT1 functions as a “Janus-headed” target: its inhibition in T cells exacerbates Th2 responses, yet its inhibition in DCs suppresses Th2-driven inflammation, underscoring the necessity for cell-specific therapeutic strategies in Th2-mediated diseases.

## Overview of SIRTs: Particularly SIRT1

Sirtuins are a family of NAD^+^-dependent deacetylases that play critical roles in cellular homeostasis by regulating metabolic pathways, aging, inflammation, and stress responses.^67^ They are highly conserved across species and consist of seven isoforms (SIRT1–SIRT7) in mammals, each with distinct cellular localization and functions.^68^ Sirtuins are involved in diverse biological processes, such as DNA repair, mitochondrial biogenesis, apoptosis, and energy metabolism.^69^^,^^70^ Their activity is closely tied to the cellular NAD^+^/NADH ratio, linking their function to metabolic states. Dysregulation of sirtuins has been implicated in various diseases, including cancer, neurodegeneration, and metabolic disorders.^71^^,^^72^^,^^73^ The discovery of SIRT1^74^ as a NAD^+^-dependent deacetylase,^75^ laid the foundation for its role in inflammation and oxidative stress. In 2003, resveratrol was identified as a SIRT1 activator,^76^ and by 2010, studies showed SIRT1’s ability to suppress airway inflammation in asthma.^77^

Studies indicate that SIRT1’s relationship with inflammation is more complex than simple anti-inflammatory effects. A recent meta-analysis (2,028 participants) found inflammatory groups exhibited significantly higher SIRT1 levels than controls, suggesting inflammation may upregulate SIRT1 as a compensatory mechanism. However, substantial heterogeneity was observed, with subgroup variations: middle-aged patients showed lower SIRT1 levels, while East Asian, autoimmune, and musculoskeletal groups displayed higher levels, indicating context-dependent roles.^78^ Another study demonstrated that cigarette smoke extract impaired the SIRT1/FOXO3 axis in bronchial epithelial cells, leading to dysregulated NF-κB activity and enhanced neutrophil chemotaxis with constitutive FOXO3 expression lower in COPD patients than controls.^79^ Collectively, these studies suggest SIRT1’s relationship with inflammation is bidirectional and context specific, varying by disease state, tissue type, and inflammatory phase supporting nuanced interpretation for SIRT1-targeted therapies.

By 2012, animal studies first linked SIRT1 deficiency to worsened COPD progression, demonstrating that cigarette smoke-induced SIRT1 reduction led to increased FOXO3 acetylation, premature cellular senescence, and alveolar cell apoptosis in mouse models.^80^ A recent double-blind randomized controlled trial evaluating resveratrol in asthma patients demonstrated improved asthma control test scores and lung function parameters alongside reduced inflammatory biomarkers.^81^^,^^82^ Comprehensive reviews now highlight SIRT1-targeted therapies for COPD exacerbations, emphasizing its dual role in anti-inflammatory and antioxidant pathways in chronic lung diseases based on accumulating clinical evidence.^83^^,^^84^^,^^85^

Based on the biological activities, SIRT1 has been extensively studied compared to other members of the SIRT family, making it a compelling candidate for asthma and COPD treatment. Its pivotal role in regulating inflammation, oxidative stress, and tissue remodeling which are the core pathological features of both condition highlights its therapeutic potential.^69^^,^^86^^,^^87^ Unlike other SIRTs, SIRT1 exhibits broad deacetylase activity, targeting key transcription factors, such as NF-κB, AP-1, and hypoxia-inducible factor (HIF-1α), which are critical in the pathophysiology of asthma and COPD.^69^^,^^88^ Additionally, SIRT1 plays a role in regulating airway smooth muscle (ASM) function, ECM remodeling, and angiogenesis, directly targeting the structural alterations and inflammation associated with these diseases.^89^^,^^90^

While other SIRTs, such as SIRT3 and SIRT6, have roles in mitochondrial function and genomic stability,^91^^,^^92^ their therapeutic scope in respiratory diseases is less defined.^93^ A recent comprehensive review summarizes the therapeutic roles of the selective SIRT1 activator SRT2104 across inflammatory conditions, metabolic disorders, neurodegenerative diseases, cardiovascular diseases, and cancer, highlighting its ability to modulate inflammation, oxidative stress, mitochondrial function, and cellular senescence with a favorable safety profile and oral bioavailability demonstrated in clinical studies.^51^ Clinical proof of mechanism for SIRT1 activation in inflammatory disease was established in a phase 2a randomized trial, where SRT2104 in moderate to severe psoriasis patients (*n* = 40) showed dose-dependent correlations between drug exposure and PASI improvement, reduced epidermal thickness, and suppressed IL-17 pathway gene expression in skin biopsies.^94^ Another preclinical study demonstrating that oral SRT2104 crossed the blood-brain barrier, extended survival, improved motor function, and reduced huntingtin aggregation in Huntington’s disease mice by enhancing SIRT1 deacetylase activity, improving mitochondrial function, and reducing oxidative stress though subsequent human trials were ultimately discontinued due to lack of efficacy.^95^ While these compounds exhibit a degree of selectivity for SIRT1, it is important to acknowledge that resveratrol, in particular, is a multi-target molecule with complex mechanisms of action, which may involve off-target effects. Nonetheless, the overall therapeutic potential and relatively favorable safety profiles of these activators support their continued investigation for clinical application. This focused review on SIRT1 allows us to delve deeply into its mechanistic pathways and therapeutic potential, paving the way for targeted interventions in asthma and COPD. There are several therapeutic applications of SIRT1 have been reported against different disorders,^51^^,^^69^^,^^96^^,^^97^^,^^98^^,^^99^^,^^100^^,^^101^ and SIRT1 offers potential in asthma and COPD by targeting inflammation and AR. Therefore, exploring inflammatory (Th2) and AR pathways are crucial for developing symptom-specific treatment strategies.

### Heterogeneity of asthma phenotypes/endotypes

Asthma is not a single disease but a spectrum of subtypes. Th2-high asthma (e.g., eosinophilic, allergic) and Th2-low asthma (e.g., neutrophilic, obesity associated) respond differently to biologics like anti-IL-5 or anti-IgE.^102^ SIRT1’s pleiotropic effects such as deacetylating NF-κB or FOXO3 may yield variable outcomes across subtypes. For instance, SIRT1 activation could suppress eosinophilia in Th2-high asthma but exacerbate neutrophilic inflammation in Th2-low subsets.^103^ Current trials lack stratification by biomarkers (e.g., fractional exhaled nitric oxide, blood eosinophils), which is critical given the failure of “one-size-fits-all” approaches in asthma.^104^

### SIRT1 in inflammation and immunity

#### SIRT1 as a regulator of inflammation

SIRT1 is pivotal in regulating inflammation by targeting transcription factors. By deacetylating the p65 subunit of NF-κB, SIRT1 reduces the expression of pro-inflammatory cytokines, such as IL-6, TNF-α, and IL-1β, mitigating chronic inflammation.^69^^,^^105^^,^^106^ SIRT1 is a potential therapeutic target in asthma and COPD by regulating inflammation and immune cell function, including deacetylating AP-1 in macrophages to reduce pro-inflammatory cytokines and oxidative stress.^107^ It has also been observed that SIRT1 negatively regulates signal transducer and activator of transcription 3 (STAT3 ), by deacetylating it, reducing its transcriptional activity. Since STAT3 mediates cytokine and growth factor responses to promote cell survival, proliferation, and differentiation, its dysregulation is implicated in chronic inflammatory diseases and cancer.^51^^,^^108^^,^^109^ This interaction is significant in controlling inflammation and immune responses, making the SIRT1-STAT3 axis a promising therapeutic target in diseases such as asthma, COPD, and other inflammatory disorders.

It has also been reported that SIRT1-NLRP3 pathway plays a key role in regulating inflammation. NLRP3 activation forms the inflammasome, promoting IL-1β and IL-18 release via caspase-1, triggering inflammation. SIRT1 suppresses this response by modulating NF-κB and nuclear factor erythroid 2 related factor 2 (Nrf2) pathways, highlighting its therapeutic relevance in inflammation-related conditions.^110^ Moreover, PPARγ and SIRT1 collaboratively promote M2 macrophage polarization, a key anti-inflammatory state. PPARγ, activated by IL-4/IL-13 via STAT6, upregulates M2-associated genes, while SIRT1 enhances this process through transcriptional regulation. Their interaction contributes to immune modulation in allergic diseases, including asthma, by promoting MRC1-mediated macrophage alternative activation.^111^

Furthermore, SIRT1 interacts with the HIF-1α pathway, which is associated with the cellular response to hypoxia.^112^ Hypoxia can worsen inflammation and is a condition often present in chronic respiratory diseases due to compromised airway function. By regulating HIF-1α activity, SIRT1 can limit hypoxia-induced inflammatory signaling, further contributing to its anti-inflammatory properties.^113^ Moreover, SIRT1 enhances glucocorticoid receptor activity via deacetylation, restoring steroid sensitivity in resistant asthma/COPD and preserving lung integrity by reducing inflammation and oxidative damage.^114^^,^^115^ Overall, the ability of SIRT1 to modulate these pathways underscores its significance as a therapeutic target, as it acts on multiple levels to suppress inflammation in the respiratory system.

#### Antioxidant mechanisms of SIRT1

SIRT1 protects against oxidative stress by enhancing mitochondrial function and antioxidant defenses.^116^ It activates PGC-1α through deacetylation, promoting mitochondrial biogenesis, ATP production, and reduced ROS generation. SIRT1 also upregulates antioxidant enzymes like superoxide dismutase (SOD) and catalase, maintaining redox balance and preventing oxidative damage to cellular components.^117^ Additionally, SIRT1 activates Nrf2 via deacetylation, enhancing its nuclear translocation and antioxidant gene expression (HO-1, SOD, GPx) to mitigate oxidative stress and ROS-induced damage.^118^ These actions mitigate ROS-induced inflammation and AR, highlighting SIRT1’s therapeutic potential in asthma and COPD management.

Moreover, SIRT1 deacetylates FOXO factors (FOXO1/3/4), enhancing their DNA binding and upregulation of antioxidant enzymes (catalase, SOD, GPx) to neutralize ROS and protect against oxidative damage.^119^ SIRT1 deacetylates p53 to prevent excessive apoptosis under oxidative stress, maintaining cellular homeostasis.^120^ In COPD, SIRT1 delays lung epithelial senescence by downregulating p16/p21 via suppression of miR-125a-5p/Sp1/HIF-1α signaling, protecting against smoke-induced aging and senescence-associated secretory phenotype activation.^121^^,^^122^

Additionally, SIRT1 reduces oxidative stress by inhibiting NADPH oxidase-mediated ROS production and promoting autophagy to clear damaged organelles. These actions protect against ROS-induced cellular damage, especially in cardiovascular and neurodegenerative diseases.^123^ Hence, SIRT1 regulates redox homeostasis via FOXO/PGC-1α/Nrf2 activation and NF-κB/p53/NADPH oxidase inhibition, while promoting autophagy (LC3/Beclin-1) to clear ROS-generating organelles. In COPD, dysregulated autophagy impairs inflammation resolution and tissue repair; enhancing autophagy via SIRT1 represents a promising therapeutic strategy.^124^

SIRT1’s roles in COPD provide valuable insights into its therapeutic potential. In COPD, reduced SIRT1 levels correlate with disease severity, while SIRT1 activation alleviates inflammation, oxidative stress, and senescence in animal models, demonstrating its protective effects.^47^^,^^125^ Moreover, SIRT1 regulates fibroblast activity and ECM remodeling, reducing fibrosis, and structural lung changes.^126^^,^^127^^,^^128^ SIRT1 activation holds potential to reduce AR and enhance lung function in asthma, given its role in addressing inflammation, oxidative stress, and remodeling shared with COPD.^51^ Hence, the roles of SIRT1 make it a promising target in asthma therapy, Wwhile existing evidence from COPD research supports the relevance of SIRT1 in respiratory disease. By counteracting multiple anti-inflammatory and antioxidant pathways, SIRT1 emerges as a novel and versatile therapeutic candidate for asthma treatment.^110^^,^^129^^,^^130^ Given their critical role, an in-depth analysis of these pathways is essential for improving treatment strategies for asthma and COPD patients.

### SIRT1 and the circadian clock

SIRT1 plays a pivotal role in regulating the circadian clock, a biological system that orchestrates physiological and metabolic rhythms based on environmental cues like light-dark cycles and feeding patterns.^131^ SIRT1 regulates the circadian clock by deacetylating core components like BMAL1 and PER2, enhancing their stability and transcriptional activity. This modulation fine-tunes CLOCK-BMAL1 complexes and alters chromatin structure, ensuring precise expression of circadian genes and tight coordination between metabolic processes and circadian rhythms.^131^^,^^132^^,^^133^^,^^134^^,^^135^^,^^136^ SIRT1 activity is coupled to circadian NAD^+^ fluctuations, forming a bidirectional feedback loop that links metabolic state with circadian rhythms and energy homeostasis.^135^^,^^136^^,^^137^^,^^138^ Moreover, SIRT1 regulates the circadian clock by deacetylating histones and clock proteins like CLOCK and BMAL1, influencing rhythmic gene expression. It modulates CLOCK:BMAL1 complexes and chromatin remodeling to ensure coordinated clock gene transcription.^136^ It has also been reported that SIRT1 links the circadian clock and metabolism by regulating metabolic gene expression in sync with the light-dark cycle. It influences glucose, lipid metabolism, and mitochondrial function while modulating PPARγ and PGC-1α activity, integrating circadian and metabolic pathways.^139^

Moreover, aging reduces NAD^+^ levels, impairing SIRT1 activity and disrupting circadian rhythms. Restoring SIRT1 improves circadian regulation and delays metabolic decline, highlighting its therapeutic potential. In the suprachiasmatic nucleus (SCN), SIRT1 regulates core CLOCK genes and enhances light responsiveness, ensuring synchronization with environmental cycles and maintaining circadian stability.^135^ Beyond the SCN, peripheral tissues, such as the liver, muscle, and adipose tissue have their own circadian rhythms. SIRT1 helps synchronize these peripheral clocks with feeding and fasting cycles, ensuring proper metabolic regulation. This coordination is essential for maintaining overall physiological homeostasis.^140^ By modulating the central and peripheral clocks, SIRT1 ensures that physiological processes are synchronized with the external environment, highlighting its importance in maintaining circadian and metabolic homeostasis.

Hence, dysregulation of SIRT1 or circadian clock components has been implicated in various pathological conditions, including metabolic disorders, cardiovascular diseases, and respiratory conditions such as asthma and COPD.^141^^,^^142^ Circadian disruptions in asthma and COPD worsen inflammation and treatment efficacy. SIRT1 modulates both circadian and metabolic pathways, making it a key target for chronotherapeutic strategies in respiratory disease.^142^^,^^143^ The therapeutic potential of targeting SIRT1 in circadian regulation lies in its ability to restore rhythm synchronization, mitigate inflammation, and address metabolic dysfunctions.

#### Chronotherapy: Harnessing circadian rhythms for treatment

Chronotherapy aligns treatment with circadian rhythms to optimize efficacy and minimize adverse effects.^144^ The circadian clock, governed by CLOCK genes and environmental cues, regulates physiological processes like hormone release and immune responses. In asthma and COPD, it influences symptom timing and severity, including airway inflammation, AHR, and nocturnal exacerbations, driven by fluctuations in cortisol, melatonin, and inflammatory mediators.^141^^,^^145^^,^^146^ Asthma symptoms often worsen at night due to circadian declines in airway patency and heightened inflammation. Similarly, COPD patients frequently experience morning dyspnea, reflecting circadian variations in lung function and oxidative stress.^147^^,^^148^ Understanding these temporal patterns provides a framework for optimizing the timing of therapeutic interventions to better target disease mechanisms when they are most active.

**Figure 1:**
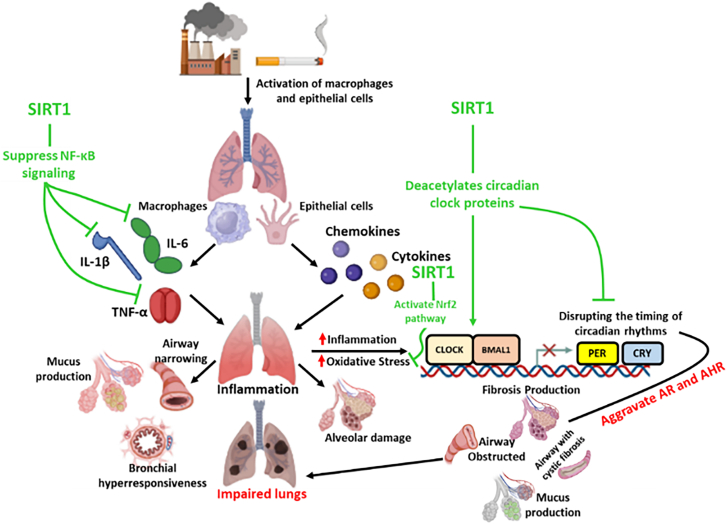
Role of SIRT1 in mitigating lung inflammation and circadian clock

Hence, chronotherapy optimizes asthma and COPD treatment by aligning corticosteroids with morning cortisol peaks, bronchodilators with nighttime bronchoconstriction, and leukotriene antagonists with overnight inflammation.^149^^,^^150^ So, advancing research in circadian biology and pharmacology is vital to integrate this strategy into clinical practice. SIRT1’s role in circadian regulation presents a promising avenue for chronotherapy in managing asthma and COPD. The circadian clock influences a variety of pathophysiological processes, which exhibit distinct daily fluctuations.^135^ SIRT1 interacts with core clock components like CLOCK and BMAL1, deacetylating them to maintain circadian rhythm stability and synchronizing metabolic and cellular processes as shown in Figure 1.

Environmental pollutants and tobacco smoke activate macrophages and epithelial cells, leading to the release of pro-inflammatory cytokines (e.g., IL-1β, IL-6, andTNF-α) and chemokines, which drive oxidative stress, inflammation, and airway narrowing. This cascade contributes to bronchial hyperresponsiveness, alveolar damage, and impaired lung function. SIRT1 mitigates these effects by suppressing NF-κB signaling, reducing cytokine production, and activating the Nrf2 pathway to enhance antioxidant defenses. Additionally, SIRT1 deacetylates circadian clock proteins (CLOCK and BMAL1), restoring circadian rhythm and ensuring proper transcription of genes like PER and CRY. Collectively, SIRT1’s actions improve lung function, reduce inflammation, AR, AHR, and prevent disease exacerbations, underscoring its therapeutic potential in asthma, COPD, and related conditions (Created with BioRender and Microsoft PowerPoint).

Activating SIRT1 during specific circadian phases could enhance its anti-inflammatory and antioxidant functions, targeting periods of heightened disease activity, such as nocturnal exacerbations or early-morning airway constriction.^47^^,^^151^^,^^152^ Despite its promise, challenges such as individual variability in circadian timing and the complexity of multi-oscillator systems must be addressed to fully realize the benefits of chronotherapy. Studies on SIRT1 activators, including resveratrol and SRT2104, underscore their therapeutic potential when administered in alignment with circadian rhythms.^51^ Timing treatment during peak inflammation or oxidative stress may enhance efficacy and reduce side effects. Integrating chronotherapy with SIRT1-targeted approaches offers a promising strategy for managing respiratory diseases.

## SIRT1 mediates AR in asthma and COPD

### Regulation of ASMCs

ASM cells (ASMCs) are central to asthma and COPD pathogenesis, driving AR, AHR, and airflow obstruction. In asthma, ASMC hyperproliferation and contractility cause airway wall thickening and excessive bronchoconstriction. In COPD, ASMCs contribute to fixed obstruction via fibrosis and ECM deposition. Their dual role in inflammation and remodeling positions ASMCs as a key therapeutic target in both diseases.^13^^,^^153^

SIRT1 deacetylase activity regulates ASMC proliferation and contractility by inhibiting NF-κB and AP-1 pathways, reducing pro-inflammatory cytokine release and limiting inflammation-induced remodeling.^89^^,^^90^ Beyond its anti-inflammatory effects, SIRT1 directly impacts structural remodeling by downregulating TGF-β/Smad signaling, a key driver of ASMC hyperplasia and ECM deposition. This modulation not only limits ASMC hyperproliferation but also reduces ECM protein accumulation, thereby preventing excessive thickening of the airway walls.^154^ Moreover, SIRT1 influences contractility by attenuating oxidative stress-induced responses in ASMCs, which are known to exacerbate bronchoconstriction in asthma and COPD. By reducing such contractile responses and oxidative damage, SIRT1 offers a multifaceted approach to mitigating AR and AHR in these respiratory diseases.^155^^,^^156^ Hence, SIRT1 approaches could lead to better control of airway obstruction and progression in patients with severe or refractory asthma and COPD.

### Impact on ECM and fibrosis

**Figure 2:**
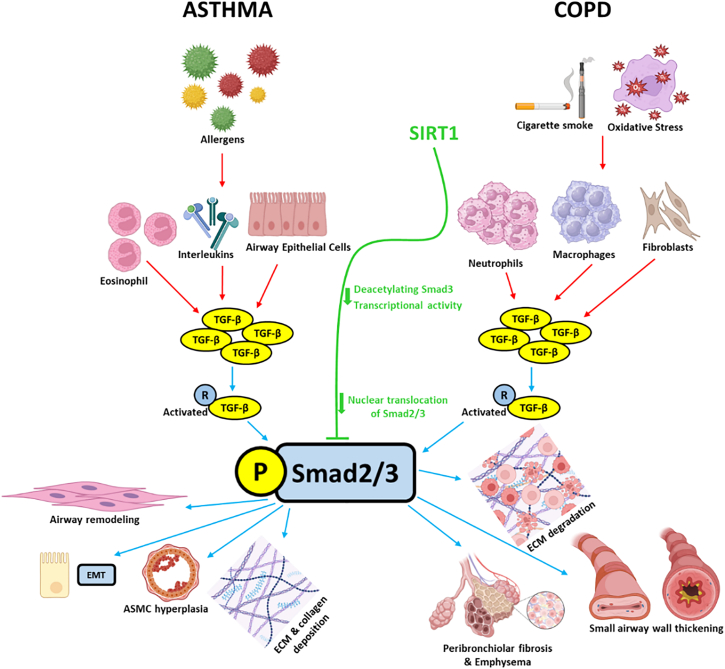
SIRT1-mediated inhibition of TGF-β/Smad signaling in airway structural remodeling in asthma and COPD

AR in asthma and COPD is characterized by significant changes in the ECM, including increased collagen deposition, fibroblast activation, and the development of fibrosis, which collectively contribute to disease progression and impaired lung function.^14^^,^^157^ SIRT1, exerts its anti-fibrotic effects by modulating ECM protein expression and fibroblast activity, thereby influencing the balance between matrix production and degradation. Specifically, SIRT1 enhances the activity of matrix metalloproteinases (MMPs), enzymes responsible for ECM degradation, while maintaining equilibrium with tissue inhibitors of metalloproteinases (TIMPs) to ensure proper ECM turnover and prevent excessive fibrosis.^158^^,^^159^ SIRT1’s antioxidant function limits oxidative stress, a key driver of fibroblast activation and ECM deposition. By mitigating oxidative damage, SIRT1 prevents fibroblast proliferation and excessive ECM accumulation, reducing tissue stiffness and airway narrowing. This protective role helps preserve airway structure and function.^160^^,^^161^ SIRT1 limits AR by downregulating the TGF-β/Smad pathway, reducing ECM deposition and fibrosis in asthma and COPD^162^^,^^163^^,^^164^ as shown in Figure 2.

In asthma, eosinophils, interleukins, and epithelial cells release TGF-β in response to allergens. In COPD, macrophages, neutrophils, fibroblasts, and epithelial cells release TGF-β following cigarette smoke exposure and oxidative stress. TGF-β binds to its receptors, phosphorylating Smad2/3 and translocate to the nucleus to drive profibrotic gene transcription. This leads to asthma-specific outcomes (AR, ECM/EMT and collagen deposition, and ASM hyperplasia) and COPD-specific outcomes (peribronchiolar fibrosis, small airway thickening, and emphysema). SIRT1 deacetylates Smad3, reducing its nuclear translocation and transcriptional activity, thereby attenuating TGF-β-driven remodeling in both diseases. This comparative view highlights shared and distinct SIRT1-sensitive pathways, supporting its therapeutic potential across chronic respiratory diseases (Created with BioRender and Microsoft PowerPoint).

Hence, SIRT1 is a promising target for treating AR and fibrosis in asthma and COPD, offering dual benefits of limiting disease progression and preserving lung function. This highlights the potential of SIRT1-based therapies to improve outcomes in patients with chronic airway diseases.

### Angiogenesis and SIRT1

SIRT1 regulates pathological angiogenesis in AR by deacetylating HIF-1α, reducing vascular endothelial growth factor (VEGF) expression and curbing excessive vascularization in asthma and COPD.^165^

SIRT1 protects endothelial cells from oxidative stress, reducing abnormal angiogenesis and vascular dysfunction. This antioxidant action helps limit airway inflammation and remodeling by preserving vascular integrity.^166^^,^^167^ Its activation offers a multi-targeted approach to reduce inflammation, oxidative stress, and structural changes in the airways. This could help alleviate symptoms and slow disease progression. Furthermore, Table 1 outlines the limited but emerging data on SIRT1’s role in treating these chronic respiratory diseases.

**Table 1:** Reported data on SIRT1 for asthma and COPD therapy

Key findings	Mechanism	Therapeutic implications	Reference
SIRT1 protects against emphysema by reducing cellular senescence.	SIRT1 deacetylates FOXO3, reducing senescence.	SIRT1 activation may be a strategy for COPD/emphysema.	Yao et al.^80^
Myricetin ameliorates airway inflammation and remodeling by activating SIRT1.	regulates the JNK/Smad3 pathway.	Potential therapeutic role of myricetin in asthma.	Huang et al.^168^
Bergenin reduces airway inflammation and remodeling by activating SIRT1 in macrophages.	affects NF-κB pathway and cytokine production.	bergenin could improve asthma-induced airway pathology.	Huang et al.^169^
SIRT1 suppresses allergic airway inflammation in macrophages.	involves ERK/p38 MAPK pathways.	SIRT1 activation in macrophages may be a therapeutic strategy for asthma.	Lai et al.^170^
Anthocyanins inhibit airway inflammation via the miR-138-5p/SIRT1 axis.	downregulates NF-κB pathway.	anthocyanins could be used to treat asthma by targeting SIRT1.	Liu et al.^171^
Suppression of SIRT1 increases IL-6 expression via Akt pathway activation in allergic asthma.	SIRT1 inhibition leads to increased IL-6 and Akt activation; SIRT1 activation reduces IL-6 expression.	activating SIRT1 could reduce inflammation in asthma.	Fukuda et al.^172^
Increased peripheral SIRT1 levels observed in asthma patients.	SIRT1 exerts anti-inflammatory effects, potentially beneficial in asthma.	enhancing SIRT1 activity may provide anti-inflammatory benefits.	Wang et al.^55^
SIRT1 targeted approach for virus-induced asthma exacerbations.	SIRT1 levels decrease in Th2 and non-Th2-related inflammation; targeting SIRT1 may treat exacerbations.	SIRT1 modulation could be a strategy for managing asthma exacerbations.	Fukuda et al.^173^
miR-221 targets SIRT1, contributing to airway epithelial cell injury in asthma.	miR-221 inhibits SIRT1 expression, affecting cell proliferation and apoptosis.	inhibiting miR-221 or enhancing SIRT1 could protect airway epithelial cells.	Zhang et al.^174^
SIRT1 is a negative regulator of MMP9 expression in COPD.	SIRT1 reduction leads to increased MMP9 expression, contributing to COPD pathology.	SIRT1 activation could reduce MMP9 levels and inflammation in COPD.	Nakamaru et al.^175^
SIRT1 protects against cigarette smoke-induced lung oxidative stress.	SIRT1 reduces oxidative stress via a FOXO3-dependent mechanism.	SIRT1 activation may protect against oxidative stress and inflammation in COPD.	Yao et al.^176^
SIRT1 is involved in regulating redox state and inflammation in COPD.	SIRT1 modulates oxidative stress and chronic inflammation, crucial in COPD progression.	targeting SIRT1 could address oxidative stress and inflammation in COPD.	Conti et al.^177^
Resveratrol activates SIRT1, reducing inflammation and oxidative stress in COPD.	SIRT1 activation by resveratrol upregulates PGC-1α, reducing IL-6 and IL-8 levels.	resveratrol or similar SIRT1 activators could be therapeutic in COPD.	Wang et al.^178^
SIRT1 activation reduces senescence of alveolar epithelial cells in COPD.	SIRT1 signaling decreases senescence via FOXO3a and p53 pathways.	SIRT1 activation may limit lung cell senescence and damage in COPD.	Yuan et al.^179^
SIRT1 levels are decreased in lungs of COPD patients.	SIRT1 reduction leads to increased acetylation of NF-κB, enhancing inflammation.	enhancing SIRT1 could reduce NF-κB-mediated inflammation in COPD.	Rajendrasozhan et al.^180^

Overall, SIRT1 restores corticosteroid sensitivity, reduces AHR, and slows emphysema progression by inhibiting NF-κB, activating FOXO3 and PGC-1α, and suppressing TGF-β and MMPs. Compounds like resveratrol and SRT2104 have shown promise in preclinical studies, while lifestyle changes (e.g., caloric restriction, exercise) may boost SIRT1 via NAD^+^ elevation. Though challenges like tissue-specific effects and age-related NAD^+^ decline exist, SIRT1 remains a promising target for novel respiratory therapies.^80^^,^^181^^,^^182^^,^^183^

### SIRT1 effects in asthma versus COPD: Key mechanistic differences

Although SIRT1 activation exerts protective effects in both asthma and COPD, the underlying mechanisms and pathological contexts differ substantially. In asthma, SIRT1 primarily modulates Th2-driven inflammation by deacetylating GATA3, the master transcription factor for Th2 cell differentiation, thereby decreasing IL-4 expression and suppressing allergic airway inflammation.^70^ Additionally, SIRT1 inhibits allergic airway inflammation by suppressing autophagy via the mTOR pathway and attenuates AR through HIF-1α/VEGF signaling.^184^ In contrast, COPD pathogenesis involves more prominent oxidative stress and cellular senescence, with SIRT1 exerting protection through distinct mechanisms: activating FOXO3a to prevent alveolar epithelial cell senescence, regulating PGC-1α/NF-κB signaling to alleviate inflammation and oxidative stress, and preserving mitochondrial function.^184^^,^^185^ This divergence reflects the fundamental pathophysiological differences; asthma is predominantly an immune-driven inflammatory disease, whereas COPD represents an “inflammaging” phenotype where chronic inflammation, premature senescence, and oxidative damage converge.^86^ Consequently, SIRT1 activators in asthma may be most beneficial for suppressing type-2 inflammation and steroid resistance, while in COPD, therapeutic strategies should prioritize mitigating oxidative stress, cellular senescence, and emphysema progression.

### Conflicting evidence and context-dependent caveats

Despite the predominant view of SIRT1 as a protective molecule, conflicting evidence necessitates nuanced interpretation. In asthma, while most studies report decreased SIRT1 expression in lung tissues and bronchial epithelial cells, serum SIRT1 levels have been found to be increased and negatively correlated with pulmonary function in some reports, whereas other studies identified no correlation or differences between disease severities.^70^ These discrepancies may reflect tissue-specific expression patterns, protein instability, or confounding factors such as age and corticosteroid use.^70^ More importantly, the role of SIRT1 in asthma remains controversial because SIRT1 exhibits cell-type specific functions: it protects against airway inflammation in epithelial cells but may exert pro-inflammatory effects in DCs, and SIRT7 actually promotes airway smooth muscle proliferation and migration, potentially aggravating remodeling.^67^^,^^70^ In COPD, while SIRT1 activation consistently shows protective effects in preclinical models, the extensive heterogeneity of COPD endotypes, including emphysema-predominant versus airway-predominant phenotypes, and varying degrees of neutrophilic inflammation, means that SIRT1-targeted therapies may not benefit all patients equally.^185^ These caveats underscore the need for careful patient stratification based on disease endotype, inflammatory profile, and tissue-specific SIRT1 expression patterns when considering therapeutic interventions.

## Gaps in clinical research

### Over-reliance on preclinical models

Preclinical models reveal SIRT1’s protective role in asthma and COPD but fall short in mimicking the chronic, complex nature of human disease, as murine asthma models typically reflect only acute, not long-term, inflammation.^186^ Additionally, interspecies differences in SIRT1 expression and function exist; human airway epithelial cells exhibit distinct redox regulation compared to rodents, potentially altering therapeutic responses.^187^ Environmental factors (e.g., pollution, viral infections) and comorbidities (e.g., obesity, gastroesophageal reflux disease) further complicate human disease but are seldom modeled preclinically.^188^ Thus, while preclinical data highlight SIRT1’s potential, translational relevance remains uncertain.

### Uncertain long-term safety and efficacy

Existing trials of SIRT1 activators (e.g., resveratrol, SRT2104) run ≤12 weeks, too brief for lifelong diseases like asthma or COPD. Long-term benefits and risks of sustained SIRT1 activation are still unknown.^189^ Continuous stimulation could disrupt cellular homeostasis, especially in the constantly repairing airway epithelium, and high-dose resveratrol can inhibit cytochrome P450 enzymes, altering corticosteroid or bronchodilator metabolism.^190^ Prolonged activation might also dampen protective immune responses or trigger fibrosis in vulnerable patients. Rigorous, extended trials are therefore essential to clarify risks of immunosuppression, metabolic imbalance, and cardiovascular events before broad implementation of SIRT1-based therapies.

### Recommendations to address gaps

To bridge these gaps, a multifaceted approach is required. First, phenotype-driven trials should incorporate biomarkers like periostin or IL-6 to stratify asthma and COPD patients into specific endotypes, ensuring that SIRT1 activators are tested in populations most likely to benefit.^191^ This precision medicine strategy could mitigate heterogeneity-related inefficiencies observed in past trials. Second, longitudinal studies spanning several years, including extension phases of existing NCT-registered trials, are essential to monitor long-term safety and assess whether SIRT1 activation modifies disease progression or prevents exacerbations. Third, exploring combination therapies such as pairing SIRT1 activators with corticosteroids or biologics could enhance efficacy through synergistic mechanisms, potentially reducing reliance on high-dose steroids.^192^ Finally, leveraging real-world evidence from registries like the CHRONICLE study for severe asthma could provide insights into practical outcomes, including adherence, cost effectiveness, and quality of life, which are often underrepresented in controlled trials.^193^ Together, these strategies would strengthen the evidence base for SIRT1-targeted therapies while addressing the unique challenges posed by chronic respiratory diseases.

Clinical data on SIRT1 activators in asthma and COPD are scarce; evidence is mostly from cell and animal models.^70^^,^^194^ Few clinical trials target asthma directly, and COPD studies offer limited insights due to pathophysiological differences.^195^^,^^196^^,^^197^^,^^198^ Given the phenotypic heterogeneity across asthma and COPD, personalized SIRT1-targeted therapeutic approaches are warranted. However, clinical trial data for SIRT1 activators remain limited and are presented in Table 2.

**Table 2:** Structured list of SIRT1 activators tested in human, with outcomes

Compound	Indication	Phase	Key outcomes	Reference
SRT2104	healthy volunteers	phase 1	well tolerated; dose-dependent but sub-proportional exposure; mean bioavailability ∼14%; significant food effect (up to 4-fold exposure increase); Tmax 1–3 h; half-life 12–20 h	Hoffmann et al.^199^
SRT2104	elderly volunteers	phase 1	safe and well tolerated; reduced cholesterol, LDL, and triglycerides; trends toward improved mitochondrial oxidative phosphorylation (31P MRS); no significant changes in oral glucose tolerance tests (OGTT) responses	Libri et al.^200^
SRT2104	type 2 diabetes	phase 2	no consistent dose-related improvements in glucose or insulin; high inter-subject pharmacokinetics variability; some improvement in lipid profiles	Baksi et al.^201^
SRT2104	psoriasis	phase 2a	primary endpoint (PASI score) not met; dose-dependent correlation between exposure and improvement; reduced epidermal thickness, and IL-17 pathway gene expression	Krueger et al.^94^
SRT2104	ulcerative colitis	phase 2	no significant efficacy; detectable colonic exposure confirmed	Sands et al.^202^
SRT2104	arterial stiffness (smokers/T2DM)	phase 2	significant reduction in augmentation pressure; trends toward improved augmentation index; no change in pulse wave velocity	Venkatasubramanian et al.^203^
Resveratrol	various (metabolic, inflammatory)	multiple	poor bioavailability; rapid metabolism; off-target effects; limited by low aqueous solubility	Hoffmann et al.^199^

### Pharmacokinetics, tissue penetration, and safety limits of SIRT1 activators

#### Pharmacokinetic challenges

The most significant limitation across SIRT1 activator trials is suboptimal and unpredictable pharmacokinetics. SRT2104 demonstrates dose-dependent but sub-proportional exposure increases, meaning doubling the dose does not proportionally increase drug levels in circulation.^199^^,^^201^ Mean oral bioavailability is only ∼14%, with substantial inter-subject variability that confounds efficacy interpretation. A notable food effect (up to 4-fold exposure increase) was observed, which, while potentially useful for maximizing exposure, introduces additional dosing complexity.^199^ For resveratrol, the pharmacokinetic limitations are even more severe like low aqueous solubility, rapid metabolism, and extremely low inherent bioavailability render it unsuitable as a selective SIRT1-targeted drug.^199^^,^^204^ Structural analogs with improved permeability are under preclinical investigation but have not yet reached clinical testing.^204^

#### Tissue penetration

Although SRT2104 can achieve detectable levels in target tissues such as colonic mucosa,^199^ data on lung tissue penetration specifically are lacking, a critical gap for respiratory disease applications. The compound crosses the blood-brain barrier in preclinical models,^51^ suggesting potential for central compartment penetration, but pulmonary distribution studies in humans have not been reported.

#### Safety profile

Across all phase 1 and 2 trials, SRT2104 demonstrated a favorable safety profile with no serious adverse reactions attributable to the drug.^199^^,^^200^^,^^201^ This consistent safety signal is encouraging for chronic dosing scenarios. However, long-term safety data beyond 28–84 days are unavailable.

Despite compelling preclinical evidence, the clinical implementation of SIRT1 activation for chronic lung disease faces several interconnected challenges. First, a pharmacokinetic-pharmacodynamic disconnect exists: human trials show high inter-subject variability and sub-proportional exposure, preventing therapeutically relevant lung concentrations, unlike controlled rodent dosing.^201^ Second, disease complexity and pathway redundancy allow compensatory inflammatory mechanisms to bypass SIRT1-mediated suppression, as seen in psoriasis trials where target engagement (reduced IL-17 expression) did not yield clinical efficacy.^199^ Third, timing and chronicity differ: preclinical studies use preventive dosing, whereas clinical trials enroll patients with established disease where structural changes (fibrosis, hypertrophy) may be irreversible.^201^^,^^203^ Fourth, cell-type specific effects complicate systemic activation, as SIRT1 exerts opposing roles (anti-inflammatory in epithelial cells but pro-inflammatory in DCs) potentially diluting benefit in the complex lung microenvironment.^199^ Finally, dose-limiting formulations force reliance on unpredictable food effects rather than achieving predictable, dose-proportional lung concentrations for precision dosing.^199^^,^^204^ Successful translation requires next-generation compounds with improved bioavailability, lung-specific distribution, and trials in earlier disease stages.

**Figure 3:**
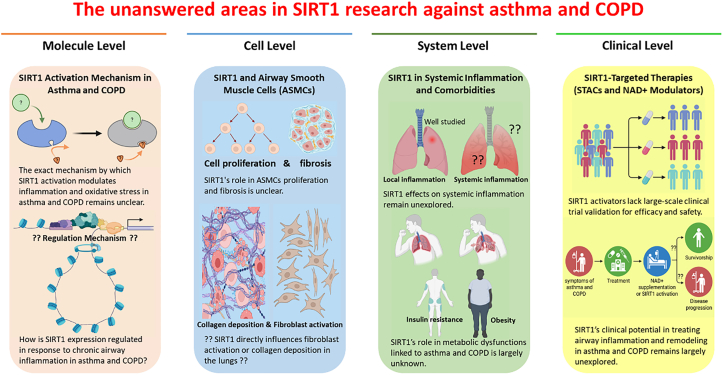
Unanswered areas in SIRT1 research for the management of asthma and COPD (Created with BioRender and Microsoft PowerPoint)

However, the long-term safety and efficacy of such interventions remain unestablished. Thus, comprehensive investigations at the molecular, cellular, systemic, and clinical levels are critically required, as outlined in Figure 3.

### Questions for future research

Future investigations should focus on translating the therapeutic potential of SIRT1 modulation into clinical practice by addressing several critical questions. A primary goal will be to determine how selective SIRT1 modulators can be developed to achieve therapeutic specificity while minimizing off-target effects, which requires a deeper understanding of the cell- and tissue-specific roles of SIRT1 in regulating both airway inflammation and structural remodeling. It remains to be seen whether SIRT1 activation can reverse established structural airway changes in chronic disease, or if its benefits are primarily limited to preventing further deterioration. To facilitate personalized approaches, research must identify reliable biomarkers that can predict which patients are most likely to respond to SIRT1-targeted interventions. Ultimately, longitudinal studies are essential to evaluate how the long-term effects of SIRT1 modulation impact disease progression and overall safety in real-world clinical settings.

## Conclusion

SIRT1 is a promising therapeutic target for asthma and COPD, offering a multifaceted strategy to combat their complex yet distinct pathophysiologies. It suppresses chronic inflammation by modulating NF-κB and STAT3 pathways, while its antioxidant effects alleviate oxidative stress, protecting against epithelial damage and immune imbalance. In asthma, SIRT1 attenuates Th2-driven inflammation, mucus hypersecretion, and ASM hyperplasia; in COPD, it reduces neutrophilic inflammation, alveolar senescence, and protease/antiprotease imbalance. Additionally, SIRT1 regulates ASM proliferation and ECM remodeling-key factors in airway fibrosis and wall thickening shared across both diseases. Preclinical studies indicate that SIRT1 activation may not only halt but potentially reverse structural changes, shifting treatment goals toward disease modification rather than symptom control alone. Translating SIRT1’s potential into clinical use requires deeper investigation into its cell-specific mechanisms and contextual differences between asthma and COPD. Despite promising animal data, clinical trials with next-generation selective SIRT1 activators (optimized for lung bioavailability and minimal off-target effects) are urgently needed.

Biomarker-guided approaches (e.g., eosinophils for asthma; oxidative stress/senescence markers for COPD) could identify endotypes most likely to benefit from SIRT1’s anti-inflammatory and antioxidant effects. Combining SIRT1 activators with existing therapies may enhance efficacy and enable steroid-sparing strategies. Ultimately, by targeting root disease mechanisms, SIRT1-based therapies offer a paradigm shift toward personalized respiratory care. Realizing this potential requires integrated efforts in mechanistic research, biomarker development, and well-designed clinical trials to ensure safe, effective, and endotype-directed treatments for obstructive airway diseases.

## Acknowledgments

Support for this work includes funding from the 10.13039/501100001809National Natural Science Foundation of China (81670252 and, 81770034), Guangdong Basic and Applied Basic Foundation (2019A1515011306), and a postdoctoral start-up fund from Shunde Women and Children’s Hospital, 10.13039/100009659Guangdong Medical University, China (2024BSHQD002).

## Author contributions

Conceptualization, M.W., Y.W., and G.M.; resources, R.P. and R.C.; writing– original draft preparation, M.W.; review and editing, M.W., R.P., R.C., Y.W., and G.M.

## Declaration of interests

The authors state no conflict of interest.
